# Tuberculose chez le personnel de santé du secteur public au Burundi: fréquence et facteurs de risque

**DOI:** 10.11604/pamj.2013.16.140.3209

**Published:** 2013-12-18

**Authors:** Olivier Mukuku, Bienvenu Mukuku Ruhindiza, Alexis Kumba Mupepe, Michel Sawadogo

**Affiliations:** 1 Faculté de Médecine, Université de Lubumbashi, République Démocratique du Congo; 2 Faculté de Médecine, Université de Ngozi, Burundi; 3 Institut Supérieur des Techniques Médicales d’Uvira, République Démocratique du Congo

**Keywords:** Tuberculose, facteurs de risque, transmission, personnel de santé, Burundi, Tuberculosis, risk factors, transmission, health personnel, Burundi

## Abstract

**Introduction:**

Le but de cette étude était de déterminer la fréquence de la tuberculose (TB) chez le personnel de santé du secteur public en charge des patients tuberculeux et d’évaluer les facteurs de risque de contracter la tuberculose chez ce personnel au Burundi.

**Méthodes:**

Il s’agit d’une étude transversale à visée analytique réalisée auprès de 300 travailleurs prestant dans 30 centres de dépistage et de traitement de la TB (CDT) au Burundi du 16 octobre au 15 novembre 2012. Les paramètres sociodémographiques et professionnels ainsi que l’antécédent de vaccination BCG de travailleurs ayant été touché par la TB ont été analysé et comparé à ceux de travailleurs qui ne l’ont pas été. Le seuil de signification a été fixé à p < 0,05.

**Résultats:**

La fréquence de la TB chez le personnel de santé est de 15%. Le risque de souffrir de la TB est de près de 4 fois chez les travailleurs âgés d’au moins 50 ans (OR=3,73; 1,53-9,08), chez ceux qui n’ont jamais reçu de vaccin de BCG (OR=3,73; 1,24-11,03), chez ceux qui n’ont pas de cicatrice vaccinale de BCG (OR=3,80; 1,67-8,62) et chez ceux qui travaillent depuis au moins 6 ans dans un CDT (OR=3,79; 1,44-9,96); ce risque est de 9 fois chez ceux qui sont mariés (OR=9,42; 1,26-70,23), de 8 fois chez ceux qui n’aèrent pas leurs salles de travail (OR=8,20; 1,48-48,23) et de 6 fois chez ceux qui ont comme profession nettoyeur ou aide-soignant (OR=6,12; 2,92-12,82). Par contre, aucune corrélation statistiquement significative n’a été observée entre le fait de souffrir de la TB et le sexe mais aussi le nombre d’heures de contact d’un travailleur avec un patient tuberculeux (p>0,05).

**Conclusion:**

L’âge, l’antécédent de vaccination de BCG ainsi que la majorité de paramètres professionnels sont en association avec la maladie TB des travailleurs de CDT. D’où, la maîtrise de certains facteurs de risque s’avère important pour faire face au fardeau de la TB parmi le personnel hospitalier.

## Introduction

La tuberculose est l’une des maladies dues à un agent infectieux unique les plus meurtrières au monde; elle se situe en seconde position juste après le VIH/Sida. Selon le Rapport Global de l’OMS sur la tuberculose publié en 2012, 8,7 millions de personnes ont développé la tuberculose et 1,4 million en sont mortes au cours de l’année 2011. Plus de 95% de ces décès se produisent dans les pays à revenu faible et intermédiaire [1]. Près d’un tiers de la population mondiale est actuellement atteint de tuberculose latente, ce qui signifie que les personnes ont été infectées par la bactérie de la tuberculose mais n’ont pas (encore) développé la maladie et ne peuvent donc pas la transmettre [2]. Des études menées dans certains pays africains et dans d’autres parties du monde ont démontré que le personnel hospitalier court un risque plus important d’infections par le *Mycobacterium tuberculosis* et de maladie tuberculeuse que la population générale [3–5]. Au Burundi, aucune étude n’a été consacrée aux enquêtes de fréquence de la tuberculose chez le personnel en charge du dépistage et du traitement des patients tuberculeux.

Notre étude se propose de déterminer la fréquence de la TB chez le personnel de santé du secteur public en charge des patients tuberculeux et d’évaluer les facteurs de risque de contracter la tuberculose chez ce personnel au Burundi.

## Méthodes

Nous avons réalisé une étude transversale à visée analytique du 16 octobre au 15 novembre 2012. Elle a été menée dans 20 centres de dépistage de TB (CDT) indépendants et dans 10 autres CDT implantés dans 10 hôpitaux différents. Ces 30 CDT sont répartis dans 14 provinces de la République du Burundi. Il s’agit de centres de référence chargés de dépistage et de traitement des malades tuberculeux au Burundi et ayant une moyenne élevée de nombre de patients tuberculeux dans leurs provinces respectives.

Seuls les travailleurs de services à risque (c’est-à-dire ceux travaillant dans la zone où le patient reçoit le traitement, le cabinet de consultation, la salle d’hospitalisations, la salle d’urgences et le laboratoire d′examen de crachat), qui avaient déjà presté plus de 2 ans dans le CDT et qui avaient accepté librement de participer à l’enquête ont été recrutés et ont fait l’objet de notre étude. Nous avons enquêté sur 3 groupes de personnel travaillant dans ces services: les soignants: médecin, infirmier, aide-soignant; les laborantins travaillant dans le laboratoire de dépistage de TB; les nettoyeurs ou ouvriers employés pour la propreté dans les locaux de services de prise en charge de la TB.

Nous avons pu retenir au total 300 agents de santé dont 38 médecins, 165 infirmiers, 57 laborantins, 13 aides-soignants et 27 nettoyeurs.

L’enquête et l’analyse documentaire ont été utilisées comme méthode. Dans chaque CDT choisi, un entretien a lieu avec le personnel sur base des questionnaires préétablis pour le recueil des données personnelles. Nous avons défini un cas de TB dans le personnel comme tout travailleur de ces CDT choisis chez qui on a diagnostiqué une TB et qui a été enregistré pour un traitement TB depuis leur prestation dans le CDT.

Les différentes données recueillies ont été codifiées, puis saisies à l’ordinateur. Les analyses statistiques ont été réalisées sur le logiciel Epi Info 2011 (version 7.0.8.3). Le test de Chi-carré (X^2^) corrigé de Yates ou le test exact de Fischer lorsque recommandé a été utilisé pour la comparaison des fréquences (exprimées en pourcentage). Les Odds ratio (OR) sont présentées avec un intervalle de confiance à 95%. Le seuil de signification a été fixé à p < 0,05.

Considérations éthiques: nous avons procédé par une série de démarches visant à obtenir les différentes autorisations nécessaires à la collecte des données. Par ailleurs, tous les travailleurs de CDT concernés étaient informés de l’objet de l’étude et y avaient adhéré volontairement.

## Résultats

**Fréquence:** des 300 enquêtés sélectionnés dans notre étude, 45 d’entre eux soit 15% ont eu à souffrir de la tuberculose après qu’ils aient été affectés dans le centre de dépistage et de prise en charge des tuberculeux.

**Aspects sociodémographiques (Tableau 1):** Concernant l’âge de nos enquêtés, la moyenne d’âge chez les travailleurs de CDT ayant souffert de la TB est de 41,86±12,06 ans allant de 25 à 70 ans alors qu’elle est de 35,96±7,40 ans comprise entre 20 et 62 ans chez ceux qui n’ont pas souffert de TB. La comparaison de ces deux moyennes montre une différence statistiquement significative (p=0,0000). L’optimum des enquêtés ayant fait la tuberculose se trouve dans la tranche des 60 à 70 ans, suivie de 50 et 59 ans. L’analyse statistique note une interdépendance entre l’âge et le fait d’avoir souffert de la TB (p=0,0054). Les enquêtés âgés d’au moins 50 ans courent un risque de près de 4 fois de souffrir de la TB (OR=3,73; 1,79-9,08).

**Tableau 1:** Aspects sociodémographiques et antécédent vaccinal de BCG

Paramètre	TTB+(n=45)	TTB -(n=255)	p	OR (IC_95%_)
**Age**				
<50 ans	36 (13,1%)	239 (86,9%)	-	1
≥50 ans	9 (36,0%)	16 (64,0%)	0,0054	3,73 (1,53-9,08)
**Etat-civil**				
Autres*	1 (2,6%)	45 (97,4%)	-	1
Mariés	44 (17,8%)	210 (82,2%)	0,0058	9,42 (1,26-70,23)
**Sexe**				
Féminin	19 (11,2%)	151 (88,8%)	-	1
Masculin	26 (20%)	104 (80%)	0,0502**	1,98 (1,05-3,77)
**Vaccin de BCG**				
Oui	38 (13,5%)	243 (86,5%)	-	1
Non	7 (36,8%)	12 (63,2%)	0,0132	3,73 (1,24-11,03)
**Cicatrice vaccinale de BCG**				
Présente	34 (12,6%)	235 (87,4%)	-	1
Absente	11 (35,5%)	104 (64,5%)	0,0018	3,80 (1,67-8,62)

* Célibataire, veuf, divorcé

** Non significatif; TTB -: travailleur n’ayant pas souffert de la tuberculose; TTB +: travailleur ayant souffert de la tuberculose

En ce qui concerne l’état-civil, il ressort de notre étude que 17,8% des 254 enquêtés mariés ont souffert de la TB alors que chez les autres (célibataires, veufs et divorcés), seul 1 sur 46 soit 2,6% a souffert de la TB. L’analyse statistique montre une association significative entre l’état-civil et le fait d’avoir souffert de la TB (p=0,0058); le risque de contracter la TB est multiplié par 9,42 chez les enquêtés mariés (OR=9,42; 1,26-70,23). Quant au sexe, 20% des 130 enquêtés de sexe masculin ont souffert de la TB contre 11,2% des enquêtés de sexe féminin mais l’analyse ne note pas de différence statistiquement significative (p=0,0502).

**Antécédent de vaccin BCG (Tableau 1):** Des 19 enquêtés qui n’ont jamais reçu de vaccin de BCG, 36,8% ont souffert de la TB contre 13,5% chez ceux ayant reçu le vaccin BGC. L’analyse statistique montre une association significative entre l’absence de vaccination BCG et la TB (p=0,0132) signifiant un risque multiplié par près de 4 fois pour les non vaccinés d’être atteint de la TB (OR: 3,73; 1,24-11,02).

Concernant la cicatrice vaccinale de BCG, il ressort que 35,5% des 31 enquêtés qui n’ont pas de cicatrice BCG ont souffert de la TB contre 12,6% chez ceux qui l’ont. En analysant statistiquement, l’étude relève qu’il existe une relation entre l’absence de la cicatrice vaccinale de BCG et le fait d’avoir souffert de la TB (p=0,0018) et rapporte un risque de près 4 de souffrir de la TB chez les enquêtés sans cicatrice vaccinale de BCG (OR=3,80; 1,60-8,62).

**Aspects professionnels (Tableau 2):** S’agissant de type de tâches à accomplir dans le service, nous constatons que chez les aides-soignants et nettoyeurs 42,5% ont été atteint par la TB alors que chez les autres (médecin, infirmier et laborantin) seuls 10,8% l’ont été. La comparaison de ces deux fréquences montre une relation statistiquement significative entre le type de tâches et le fait de contracter de la TB (p=0,0000012). Les aides-soignants et nettoyeurs courent un risque multiplié par 7 d’être atteints de la TB par rapport aux autres professions (OR=7,13; 3,34-15,19).

**Tableau 2:** Aspects professionnels

Paramètre	TTB+(n=45)	TTB - (n=255)	p	OR (IC_95%_)
**Type de tâches**				
Autres*	28 (10,8%)	232 (89,2%)	-	1
Aides-soignants/Nettoyeurs	17 (42,5%)	23 (57,5%)	0,000001	6,12 (2,92-12,82)
**Nombre d’années de travail**				
< 6 ans	5 (5,7%)	82 (94,30%)	-	1
≥ 6 ans	40 (18,8%)	173 (81,20%)	0,0071	3,79 (1,44-9,96)
**Temps contact avec un patient tuberculeux**				
< 6 heures	1 (5,3%)	18 (94,7%)	-	1
≥ 6 heures	44 (15,7%)	237 (84,3%)	0,3266**	3,34 (0,43-25,68)
**Aération des salles de travail**				
Toujours	41 (14,0%)	252 (86,0%)	-	1
Parfois/Jamais	4 (57,1%)	3 (42,9%)	0,0110	8,20 (1,48-48,23)

* Médecin, infirmier, laborantin

** Non significatif; TTB -: travailleur n’ayant pas souffert de la tuberculose; TTB +: travailleur ayant souffert de la tuberculose

Pour ce qui est du nombre d’années de travail, 18,8% des 213 enquêtés qui ont au moins 6 ans de travail ont souffert de la TB contre 5,7% des 87 enquêtés qui ont moins de 6 ans de travail. L’analyse statistique montre une relation significative entre le fait de contracter la TB et le nombre d’années de travail supérieur ou égal à 6 ans (p=0,0071); le risque est multiplié par 3,79 fois chez ceux qui ont réalisé au moins 6 ans de travail (OR=3,79; 1,44-9,96).

En ce qui concerne le temps de contact avec un patient tuberculeux, nous avons enregistré 15,7% de 281 enquêtés qui passent au moins 6 heures ont souffert de la TB contre 5,3% chez ceux qui passent moins de 6 heures. L’analyse statistique ne montre pas d’association entre le temps de contact avec un patient tuberculeux et le fait de contracter la TB (p=0,3266).

Des 293 enquêtés qui aèrent toujours leurs salles de travail, 41 soit 14% ont souffert de la TB contre 4 des 7 soit 57,1% chez ceux qui ne les aèrent pas. L’analyse statistique donne une différence significative en défaveur des enquêtés qui n’aèrent pas leurs salles de travail (p=0,0110) et ceux-ci présentent un risque multiplié par 8,2 fois de souffrir de la TB (OR=8,2; 1,48-48,23).

## Discussion

### Fréquence

La fréquence de la tuberculose (TB) chez les travailleurs de CDT dans notre étude est de 15% (45/300). Cette prévalence est légèrement supérieure à 9,9% enregistré par Harada au Japon et par Schablon en Allemagne [6, 7]. Par contre, elle est de loin inférieure à celles trouvées dans les études menées en Inde, en Russie, au Viet-Nam et en Géorgie rapportant respectivement 40,0%, 40,8%, 47,3% et 60,0% [8–11]. Une revue de littérature faite par Joshi en 2006 rapporte une prévalence qui varie de 33% à 79% dans les pays à faibles et moyens revenus [12].

Cette faible fréquence dans notre étude ne pourrait être attribuée à l′inclusion de diverses catégories de travailleurs qui ne courent pas le même risque d′exposition à la tuberculose en milieu de travail. Les divergences constatées s’expliqueraient par le fait que dans la plupart de ces études à forte prévalence, le diagnostic de la TB ne se limitait qu’à l’utilisation du test cutané à la tuberculine qui comporte des biais de sélection; c’est le cas de faux positifs pourrait être attribué à la vaccination par le bacille de Calmette-Guérin (BCG) et aux infections mycobactériennes non tuberculeuses [13]. Dans la nôtre, il s’agissait de répertorier les travailleurs qui ont eu à souffrir de la TB; cette dernière ayant été diagnostiquée et traitée.

### Facteurs de risque

Les travailleurs de CDT courent un risque accru d’être atteint de la maladie tuberculeuse et dans notre étude, l’analyse statistique montre que la fréquence de la TB a été significativement plus élevée chez les enquêtés plus âgés, chez ceux qui n’ont jamais reçu de vaccin de BCG, chez ceux qui ne présentent pas de cicatrice de BCG, chez ceux qui n’aèrent pas leurs salles de travail, chez ceux qui travaillent depuis plusieurs années dans le CDT et chez les nettoyeurs et aides-soignants. La littérature publiée au sujet des facteurs de risque de transmission de la TB chez le personnel soignant donne d’une part des résultats concordants et d’autre part des résultats divergents avec les nôtres:

<>Age et année de travail: Nous avons trouvé que les enquêtés âgés d’au moins 50 ans courent un risque de près de quatre fois de souffrir de la TB (p < 0,05). Nous avons noté également que le risque de maladie tuberculeuse est de près de quatre fois plus élevés pour le personnel qui ont au moins 6 ans de travail (p < 0,05). La durée de travail est étroitement liée à l′âge et il est difficile d′évaluer son effet indépendant sur la TB. Dans une étude menée auprès de 999 travailleurs chinois de soins de santé par He, un âge plus avancé et une carrière plus longue ont été en association significative avec une infection-TB [14]. Quant à l’étude menée en Inde par Pai, celle-ci établit un lien statistiquement significatif entre l’infection tuberculeuse et une longue durée de travail (≥ 10 ans) dans le CDT [8]. Ceci serait dû probablement au fait que le risque d′infection dépend principalement de la concentration de microgouttelettes infectieuses dans l′air et de la durée d′exposition à un patient bacillifère [15]. Cette durée de travail est amenée à plus de 11 ans dans l’étude de Rafiza [16].

**Etat-civil:** Notre étude relève que l’état-civil et le fait d’avoir souffert de la TB sont associés significativement et que le risque de contracter la TB est multiplié par 9,42 chez les enquêtés mariés (p < 0,05) comparativement aux autres (célibataires, veufs et divorcés). Nos résultats divergent avec ceux de Rafiza qui lui rapporte que le fait de ne pas être marié était statistiquement retrouvé de manière significative comme facteur de risque de contracter une infection-TB [16].

**Type de tâches:** S’agissant du type de tâches accomplies par les travailleurs, dans notre étude, les aides-soignants et nettoyeurs courent un risque multiplié par 7 fois d’être atteints de la TB par rapport aux autres professions (p < 0,05). Comme Galgalo [17], nous pensons également que les aides-soignants et les nettoyeurs passent autant de temps dans des locaux avec les patients que les autres et sont exposés de manière permanente au *Mycobacterium tuberculosis* présent dans l’air et peut-être un plus que les autres professions. Ce constat est presque identique à celui de Pai qui trouve aussi que la profession d’aide-soignant avait un risque élevé d’être infecté [8]. Mais, pour les auteurs tels que Jiamjarasrangsi, Rafiza et Roth, c’est le travail d’infirmier qui est en association significative avec la maladie-TB chez les travailleurs dans les contextes à haute prévalence de TB [16, 18, 19]. Contrairement à nos observations, dans une étude ougandaise faite à Kampala, les auteurs ne trouvent pas d’association significative entre le type de travail avec une infection-TB [20]. D’autres études antérieures ont aussi noté la durée et le type de travail comme facteurs de risque de contamination de l’infection-TB chez les travailleurs [8, 9, 11].

**Aération des salles de travail:** Concernant l’aération des salles de travail, nous avons trouvé l’existence d’une association significative entre le manque d’aération et le fait de souffrir de la TB (p < 0,05) et les enquêtés qui n’aèrent pas leurs salles de travail présentent un risque multiplié par 8,2 fois de souffrir de la TB. Il est reconnu qu’une ventilation adéquate est cruciale pour limiter le risque d’infection et que ce dernier augmente de façon significative lorsque le nombre de renouvellements d’air est inférieur à deux par heure [21]. Un article du Groupe de travail du Conseil Supérieur d’Hygiène Publique de France sur la Prévention de la transmission de la tuberculose en établissement de santé souligne l’importance de la ventilation naturelle, celle-ci assurée par le vent entre l’extérieur et l’intérieur et s’effectuant au moyen de l’ouverture des fenêtres ou des portes donnant sur l’extérieur [22].

**Vaccination de BCG:** Dans l’étude, les enquêtés qui n’ont jamais reçu de vaccin de BCG présentent un risque multiplié par près de quatre fois comparativement à ceux qui l’ont déjà reçu (p < 0,05). Il en est de même de ceux ne présentant pas de cicatrice vaccinale de BCG, ils ont un risque de souffrir de la maladie-TB près de quatre fois par rapport à ceux qui en ont (p < 0,05). Kang et Nienhaus rapportent aussi une association positive entre une infection-TB et la non-vaccination de BCG [23, 24].

**Autres facteurs:** D’autres paramètres tels que le sexe et le nombre d’heures de travail par jour n’ont pas été liés significativement à la TB dans notre étude. Par contre, les études de Rafiza, Haley et Koppaka rapportent une association statistiquement significative entre le sexe masculin et une tuberculose active [16, 25, 26]. Une étude réalisée en Chine constate que les travailleurs qui ont passé plus de 4 heures par jour en contact direct avec les tuberculeux avaient significativement une prévalence très élevée d’infection-TB [14]. Une étude menée au Kenya note que pour chaque heure supplémentaire passée par jour par le personnel hospitalier dans un local avec des patients tuberculeux, son risque de voir le diagnostic de TB porté chez lui est multiplié par 1,3 [17]. L’absence d’association entre ces paramètres et la TB dans notre étude pourrait être imputée à la petitesse de notre échantillon comparativement à d’autres études.

## Conclusion

La tuberculose constitue un véritable problème de santé publique au Burundi, car sa fréquence chez le personnel de santé du secteur public reste élevée. L’âge, l’antécédent de vaccination de BCG ainsi que la majorité de paramètres professionnels sont en association avec la maladie TB des travailleurs de CDT. D’où, les CDT doivent améliorer leurs pratiques de lutte contre l’infection, assurer des services de qualité pour la surveillance des maladies professionnelles et encourager le personnel à se faire tester pour l’infection tuberculeuse afin de faire face au fardeau de la TB parmi le personnel hospitalier.
